# Neural Correlates of Emotion Processing in Word Detection Task

**DOI:** 10.3389/fpsyg.2018.00832

**Published:** 2018-05-25

**Authors:** Wenshuang Zhao, Liang Chen, Chunxia Zhou, Wenbo Luo

**Affiliations:** ^1^Research Center of Brain and Cognitive Neuroscience, Liaoning Normal University, Dalian, China; ^2^School of Psychology, Southwest University, Chongqing, China; ^3^Chongqing College of Electronic Engineering, Chongqing, China; ^4^Laboratory of Emotion and Mental Health, Chongqing University of Arts and Sciences, Chongqing, China

**Keywords:** emotion processing, emotion words, word detection task, rapid serial visual presentation, event-related potential

## Abstract

In our previous study, we have proposed a three-stage model of emotion processing; in the current study, we investigated whether the ERP component may be different when the emotional content of stimuli is task-irrelevant. In this study, a dual-target rapid serial visual presentation (RSVP) task was used to investigate how the emotional content of words modulates the time course of neural dynamics. Participants performed the task in which affectively positive, negative, and neutral adjectives were rapidly presented while event-related potentials (ERPs) were recorded from 18 undergraduates. The N170 component was enhanced for negative words relative to positive and neutral words. This indicates that automatic processing of negative information occurred at an early perceptual processing stage. In addition, later brain potentials such as the late positive potential (LPP) were only enhanced for positive words in the 480–580-ms post-stimulus window, while a relatively large amplitude signal was elicited by positive and negative words between 580 and 680 ms. These results indicate that different types of emotional content are processed distinctly at different time windows of the LPP, which is in contrast with the results of studies on task-relevant emotional processing. More generally, these findings suggest that a negativity bias to negative words remains to be found in emotion-irrelevant tasks, and that the LPP component reflects dynamic separation of emotion valence.

## Introduction

Emotional stimuli can be processed preferentially and draw attention quickly. From an evolutionary perspective, preferential and quick responses to emotional stimuli are considered biologically adaptive (Schupp et al., 2006; Vuilleumier and Pourtois, 2007). Currently, more and more researchers employ affective pictures, facial expressions, and emotional words as stimuli in order to elicit emotional processes. Although symbolic cues, such as affective words, can also convey emotionally relevant information, compared to affective pictures and facial expressions, extracting emotional information from written words is slower and less direct (Schacht and Sommer, 2009a). Thus, one might expect the neural processing of emotional words to follow a time course that is different to that of the processing of emotional pictures and faces. However, a series of studies recently suggested that the neural processing elicited by affective words, pictures, and faces may, in fact, be similar (Sprengelmeyer and Jentzsch, 2006; Kissler et al., 2007). Specifically, the processing of emotional words can be divided into three stages (Zhang et al., 2014), seemed to be similar to the three-stage model of facial expression emotional processing proposed by Luo et al. (2010). The first stage where negative facial expressions are distinguished from others could be reflected by the N1 and P1 components. The vertex positive potential (VPP) and N170 may reflect the second stage, during which emotional facial stimuli is distinguished from neutral facial stimuli. Finally, in the third stage, the N3 and P3 components which respond differently to positive stimuli and negative stimuli probably reflect the further evaluation of information related to the affective valence of stimuli. If the three-stage model applies to both emotional words and facial expressions, this might indicate that the model is universal to all emotional information processing independent of the task. Our aim here was to test whether the three-stage model applies to emotion-relevant tasks only, or whether tasks with no emotional components also follow this processing structure.

Thus far, studies have provided inconsistent data concerning very early modulatory effects of emotional words. van Hooff et al. (2008) showed that affectively positive words elicit a larger P1 amplitude than negative and neutral words in an emotional Stroop task (van Hooff et al., 2008). However, others have failed to find a P1 effect using implicit and explicit tasks, namely color naming and emotion judgment tasks, respectively (Frühholz et al., 2011). We speculate that the early effect of emotional stimuli on event-related potential (ERP) emerges under particular experimental conditions. Further, lexical variables such as word frequency may interact with the emotions elicited by words (Scott et al., 2009). The present body of evidence suggests that the early ERP components around 200 ms post-stimulus, have a greater amplitude in response to emotional words in contrast to neutral words (Bernat et al., 2001; Ortigue et al., 2004; Scott et al., 2009). For example, using an emotional Stroop task in which subjects completed color-relevant tasks and word-relevant tasks, in the color-relevant tasks the color of word was identified and in the word-relevant tasks the words were classified as threatening or not, earlier research found that threat words were associated with a larger P2 amplitude across tasks (Thomas et al., 2007). Therefore, Lei et al. (2017) showed that P2 play an important role in the unconscious processing of emotional words.

Following these early effects, modulation of later time segments of ERPs by affective words has been reported, such as the N170 component, measured over the occipito-temporal regions (Williams et al., 2006). Scott et al. (2009) found that a negative-polarity ERP component which was at a similar latency to the face-sensitive N170 can be modulated by emotional words (Scott et al., 2009), and that words can elicit an N170 component comparable to that found for faces (Rossion et al., 2003). Luo et al. (2010) found that compared with neutral faces, emotional faces elicited larger N170 amplitudes. However, this component modulated by words is likely more left-lateralized than modulated by faces (Thomas et al., 2007; Mercure et al., 2008). This lateralization was also demonstrated recently by Zhang et al. (2014), who found that emotional words can modulate the N170 component and elicit larger amplitudes in the left hemisphere in response to positive and negative words, in contrast to neutral words (Zhang et al., 2014). However, the modulation of neural signals with a similar latency to the N170 by emotional words is not invariably found. For example, emotional words have failed to reveal such a modulation in a lexical decision task (LDT; Frühholz et al., 2011). Therefore, in previous studies, researchers used the relatively late N170 component, because when the task used in the RSVP paradigm is relatively difficult may result in late ERP delay (Kessler et al., 2005; Luo et al., 2013; Zhang et al., 2014). We assumed that the modulatory effect of emotional words on the N170 component may be task-dependent. If so, this further implies that the processing of emotional words is incompatible with the three-stage scheme in emotion-irrelevant tasks, in which attention is not explicitly directed toward the emotional content of the stimuli.

Although the effects of emotional stimuli on very early ERP components are still unclear, researches on the late ERP components modulated by affective words are frequently replicated (Fischler and Bradley, 2006). Regarding the late time period, late positive potential (LPP) amplitude is modulated by the meaning or value of a stimulus, and has been shown to vary systematically with the experienced intensity of the affective picture content (Cuthbert et al., 2000). First, the amplitudes of the LPP is sensitive to the valence of stimuli. For example, prior studies have found that the amplitudes of the LPP elicited by positive and negative words were significantly different (Bernat et al., 2001; Fischler and Bradley, 2006; Carretié et al., 2008; Pessoa and Adolphs, 2010; Bayer et al., 2012), indicating that LPP component can discriminate the valence of emotion. Second, most prior studies (Kissler et al., 2009) have employed negative words with arousal ratings greater than those of positive words and neutral words, since the relationship takes a U-shape curve between emotional arousal and emotional valence ratings of words. However, recent studies that have adopted emotional words with similar arousal ratings suggested that LPP modulation by cortical and subcortical structures associated with visual and emotional processing is valence specific (Liu et al., 2012). Third, the LPP emotion effect is sensitive to the semantics of the emotional word stimuli. Naumann et al. (1997) reported a larger LPP to unpleasant words emerged only when subjects directed attention to the dimension of emotion, in the study, where subjects made concrete versus abstract and unpleasant versus neutral decisions, emotional words were employed in a letter-search paradigm. Fischler and Bradley (2006) found that LPP emotion effects emerged when participants explicitly evaluated emotional content or performed other semantic tasks, and diminished in tasks involving orthographic judgments or lexical decisions (Fischler and Bradley, 2006). Thus, different meaningful stimuli may share a proportion of neural generators, and different types of task may recruit different neural structures (Kissler et al., 2009).

The rapid serial visual presentation (RSVP) paradigm is sensitive enough to investigate the time course of the neural emotion processing in the brain, when attentional resources are limited (Luo et al., 2010). It has been shown that the arousal level evoked by stimuli has a robust effect on the phase of perception and attention processing (Keil et al., 2006; Schacht and Sommer, 2009b). Still, questions remain as to whether the effects of emotional stimuli on ERPs modulated by a specific category or dimension of emotions (such as valence or arousal). Some results indicate specific effects for positive (Kissler and Koessler, 2011) or negative words (Bernat et al., 2001). To our knowledge, relatively few studies have manipulated pure valence effects (Luo et al., 2010; Bayer et al., 2012; Zhang et al., 2014). In a previous study (Luo et al., 2010; Zhang et al., 2014), we asked participants to judge the emotional content of facial expressions and emotional words. That task allowed top-down influences to potentially alter emotion processing. In the present study, we investigated whether the three-stage model of emotional processing still applies when subjects do not direct their attention explicitly to the emotional nature of the stimuli using the RSVP paradigm. To do this, we used a task in which participants engaged in emotion-irrelevant judgments while some stimuli were emotional in nature. We predicted that compared to the positive words and neutral words, negative affect words would elicit a larger N170 component, and different types of emotional content may be processed distinctly at different time windows of LPP.

## Materials and Methods

### Participants

Participants were 18 healthy undergraduate students (50% female; age range 18–24 years; mean age = 20.8 years) who received payment for their participation. All subjects were right-handed and had normal or corrected-to-normal vision. All participants were provided written informed consent prior to the experiment, which was approved by the Ethics Committee of The Liaoning Normal University. The experiment was approved by Southwest University Human Research Institutional Review Board and in accordance with the Declaration of Helsinki (1991).

### Stimuli

Eighteen Chinese adjectives, twelve Chinese pseudowords, and four strings that consist of four repeated digits served as stimuli, described in detail below. To avoid floor effect, we created the meaningless pseudowords which were presented upside-down during the experiment, since it was not easy to detect upright emotional adjectives in a rapidly displayed upright pseudowords sequence. The adjectives words were selected from the Chinese Affective Words System (CAWS; Wang and Zhou, 2008; Zhang et al., 2010), including six positive, six negative, and six neutral words. The three types of adjectives were similar in frequency of occurrence [*F*(2,15) < 1; positive = 44.83 ± 12.78 (*M* ±*SD*), neutral = 39.67 ± 9.16, negative = 38.33 ± 11.91 times per million], a moderate level of frequency in common language may enhance our discovery of an emotionally sensitive early N170. The three emotion conditions differed significantly in valence [*F*(2,15) = 378, *P* < 0.001, $\eta_{p}^{2}$ = 0.981; positive = 6.75 ± 0.18, neutral = 4.83 ± 0.34, negative = 2.87 ± 0.17] while there was no significant difference in the strokes [*F*(2,15) < 1; positive = 17.67 ± 4.59, neutral = 16.00 ± 3.29, negative = 16.33 ± 2.66]. We specifically controlled the arousal level of selected words across three emotional conditions [*F*(2,15) < 1; positive = 4.82 ± 0.32, neutral = 4.88 ± 0.41, negative = 4.84 ± 0.14] to confirm that our results were accurate and not contaminated by the arousal. The font of the characters was Song Ti No. 48. All stimuli were presented in the center of the screen and displayed in white color with black background. All stimuli were similar to another in size (142 × 74 pixels), contrast grade, brightness, and viewing angle (2.4 × 4.5°). The screen resolution was 72 pixels per inch. Participants were seated in a sound attenuated room with their eyes approximately 90 cm from a 17-inch screen.

### Procedure

**FIGURE 1:**
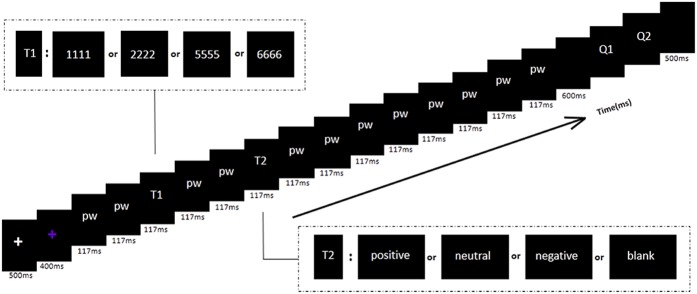
The RSVP paradigm used in this experiment. All the words were in Chinese (pseudo-words and emotional adjectives). Each trial contained twelve pseudo-words (pw), two target stimuli (T1 and T2), and two questions (Q1 and Q2). The time interval between T1 and T2 was 234 ms. The first question (Q1) asked whether the numerical T1 stimulus was odd or even. The second question (Q2) asked whether the participant had seen the T2 stimulus upright word or unseen.

As shown in **Figure 1**, at the beginning of each trial, a white fixation cross presenting for 500 ms, followed by a blue fixation cross presenting for 400 ms appeared in the center of the screen. Then a stimulus stream was presented which contained two target stimuli and twelve distracting stimuli (Chinese pseudowords). Each stimulus was presented for 117 ms.

The first target (T1) which was randomly one of the four upright digit strings appeared randomly in the third, fourth or fifth position of the stimulus series. The second target (T2) was one of the three categories of upright adjectives and a black screen, with the same occurrence probability in all conditions. T2 was presented in the third position after T1 [stimulus onset asynchrony (Pessoa and Adolphs, 2010) = 351 ms]. To get rid of the superimposed electrical activity elicited by the distractors shown before and after T2, a baseline condition was employed with a black and blank screen in place of T2 (Vogel et al., 1998; Sergent et al., 2005). This null condition enabled the ERPs elicited purely by T2 to be obtained. After each trial, subjects were presented with a question about T1 and a question about T2, participants were instructed to respond as accurately as possible, by pressing a key. The first question asked whether the numerical T1 stimulus was odd (press Key ‘1’) or even (press Key ‘2’). The second question asked whether the T2 stimulus upright word had appeared at all (press Key ‘1’ when word had appeared and Key ‘2’ when word had not appeared). The two questions were appeared on the screen serially in a fixed order at the end of each trial. There was no time limit imposed on the response, each question disappeared immediately after the subject indicated his or her response, with no feedback provided regarding response accuracy. After responding, the screen would stay blank and black for 600 ms before the next trial started (**Figure 1**). The experiment was divided into four blocks of 120 trials each. We used E-prime to program the experimental procedure.

### ERP Recording

The electroencephalogram (EEG) was recorded from 64 channels using the standard 10–20 system (Brain Products, Gilching, Germany) with an online reference to the left mastoid and an offline re-reference to the average of the left and right mastoids. The ocular artifacts of EEG data were removed by an off-line method proposed by Gratton et al. (1983). The horizontal electrooculograms (EOGs) were recorded via two electrodes positioned at the outer canthi of both eyes. Vertical EOGs were recoded via two electrodes placed above and below the right eye. Recording impedance for all electrodes was held beneath 5 kΩ. Recording bandwidth was 0.01–100 Hz, at a sampling rate of 500 Hz. Signals were re-referenced offline to obtain a global average. The EEG and EOG were amplified and followed off-line. Eye movement artifacts and trials with EOG artifacts (defined as a mean EOG voltage exceeding ± 80 μV) were automatically removed. EEG data were accepted for analysis only for trials where the response for T1 and T2 was correct. The averages were then digitally filtered (0.01–30 Hz, 24 dB/octave).

### Measures and Analysis

The averaged epoch used for ERP analysis was 1200 ms, which included a 200 ms pre -stimulus baseline. Trials were included only if the responses for T1 and T2 were correct. The valid trial number for each condition to compute average ERP waveforms were 108 trials for positive words condition, 105 trials for negative words condition and 91 trials for neutral words condition.. To get the difference between emotional condition and baseline condition, we calculated stimulus-locked average ERPs for the affectively positive, neutral, and negative word conditions of every subject. We averaged the trials by two ways, one is the average ERP for every condition and subject, the other is the average ERP for every condition across subjects (grand average). According to the topographical distribution of grand-mean ERP activity and supported by previous studies (Luo et al., 2010; Zhang et al., 2014), we used different sets of electrodes to calculate the N170 and LPP components. The N170 component mean amplitude was obtained using electrode positions P7, P8, PO7, and PO8, within a time window of 250–290 ms. Nine electrode sites (Cz, C3, C4, CPz, CP3, CP4, Pz, P3, and P4) were selected for analysis of the LPP mean amplitude, using time windows of 480–580 ms and 580–680 ms. To calculate the mean amplitude, the time windows were selected in the center of peak latencies of the ERP components in the grand-averaged waveforms. Three-way repeated measures analyses of variance (ANOVAs) were conducted. The first ANOVA used the mean amplitude of the N170 component as the dependent variable, while the second used the mean amplitude of the LPP component. Each analysis used within-subjects independent variables consisting of emotion types (three levels: positive, neutral, and negative), hemisphere (two levels for N170: right and left; three levels for LPP: right, midline, and left), and electrodes (according to different ERP components use different electrode sites as mentioned above). ANOVA *P*-values were calculated using the Greenhouse–Geisser correction. *Post hoc* pairwise tests were corrected for multiple comparisons using the Bonferroni method. The software used to analyze the behavioral and ERP data were Statistical Product and Service Solutions (SPSS) and Analyzer (Brain Product).

## Results

### Behavioral Performance

ANOVAs revealed that response accuracy was significantly affected by emotion type [*F*(2,34) = 8.29, *P* < 0.01, $\eta_{p}^{2}$ = 0.328]. Participants were significantly better at determining the presence of positive words and negative words than that of neutral words [positive = 89.06 ± 11.04% (*M* ±*SD*), *P* < 0.001; negative = 88.06 ± 9.52%, *P* < 0.05; neutral = 81.11 ± 12.24%]. However, the difference in accuracy between positive and negative words was not significant (*P* = 0.690).

### ERP Data Analysis

#### N170

**FIGURE 2:**
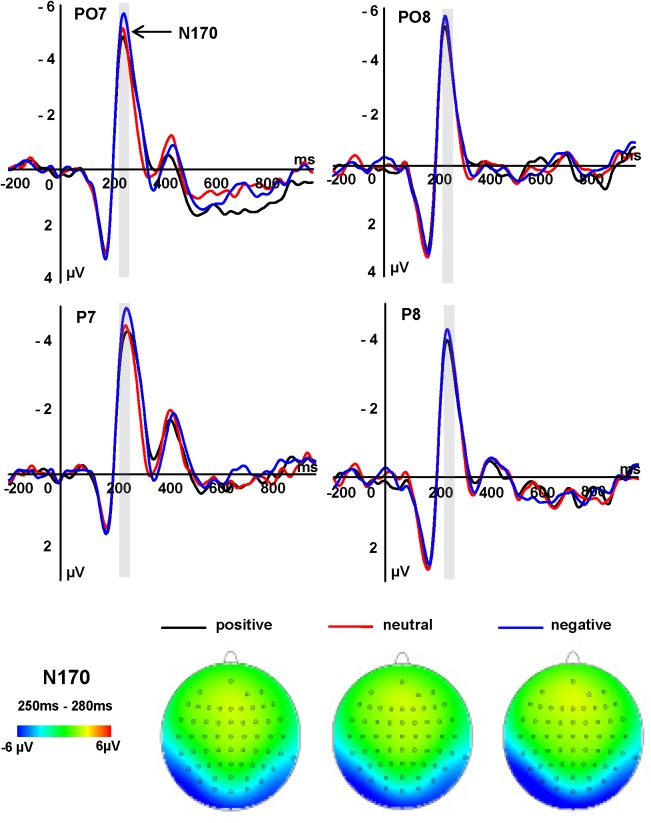
Grand average ERPs of N170 components at indicated electrode sites and topographies of the N170 component, across three conditions.

The N170 amplitudes showed significant main effects of word emotion type and electrode [*F*(2,34) = 5.84, *P* = 0.016, $\eta_{p}^{2}$ = 0.405; *F*(1,17) = 19.11, *P* < 0.001, $\eta_{p}^{2}$ = 0.529, respectively]. Negative affect words (-4.71 μV) elicited a more negative N170 amplitude than did positive words (-3.86 μV, *P* = 0.011) and neutral words (-4.03 μV, *P* = 0.047), while there was no significant difference between the latter two conditions (*P* = 0.553). PO7/8 (-4.59 μV, *P* < 0.011) electrode sites elicited larger N170 amplitudes than did P7/8 sites (-3.86 μV; **Figure 2**).

#### LPP (480–580 ms)

The LPP amplitudes revealed significant main effects of word type and hemisphere [*F*(2,34) = 4.31, *P* = 0.028, $\eta_{p}^{2}$ = 0.202; *F*(2,34) = 3.74, *P* = 0.040, $\eta_{p}^{2}$ = 0.180, respectively]. Pairwise comparisons showed that positive words (1.71 μV) elicited a larger LPP amplitude than did negative (1.29 μV, *P* = 0.034) and neutral words (1.28 μV, *P* = 0.036), while there was no significant difference between the latter two conditions (*P* = 1.000). The midline region elicited larger LPP amplitudes, on average (1.80 μV), than did the right hemisphere (1.15 μV, *P* = 0.033). There was no significant difference between right and left hemispheres (1.33 μV, *P* = 0.140), or between the left hemisphere and midline region (*P* = 1.000).

#### LPP (580–680 ms)

**FIGURE 3:**
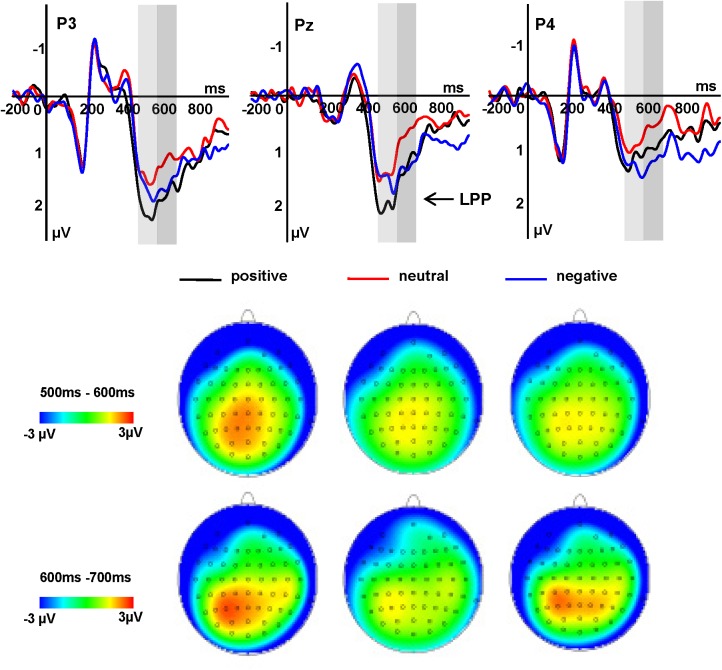
Grand average ERPs of LPP components in two different time windows at indicated electrode sites and topographies of the LPP component in two different time windows, across three conditions.

The LPP amplitudes revealed significant main effects of word type and electrode [*F*(2,34) = 4.72, *P* = 0.019, $\eta_{p}^{2}$ = 0.217; *F*(2,34) = 7.10, *P* = 0.009, $\eta_{p}^{2}$ = 0.294]. The pairwise comparison revealed that positive (1.39 μV, *P* = 0.047) and negative words (1.42 μV, *P* = 0.031) elicited larger LPP amplitudes than did neutral words (0.98 μV), while there was no significant difference between the former two conditions (*P =* 1.000). The P3/Pz/P4 electrode sites (1.63 μV, *P* = 0.035) and CP3/CPz/CP4 (1.36 μV, *P* = 0.017) sites elicited larger LPP amplitudes than did the C3/Cz/C4 (0.79 μV) sites, while there was no significant difference between the former two conditions (*P* = 0.512; **Figure 3**).

## Discussion

This study investigated the time course of emotional word processing using a dual-target RSVP task. Our behavioral data showed that the accuracy for affectively positive and negative adjectives was higher than the accuracy for neutral adjectives. Our results of ERP components support the idea that emotional stimuli are preferentially processed. Two ERP components were measured, the N170 and the LPP. Negative adjectives elicited a more negative N170 amplitude than positive and neutral adjectives. Furthermore, we analyzed the LPP in two time windows, and found the LPP amplitude elicited by positive words was larger than the amplitude for negative words and neutral words, consistent with previous results.

### Early Effects of Word Emotion Type

In the current study, we found no early modulatory effect of emotional words on the P1 component, which is in accordance with other studies on emotional word processing (Herbert et al., 2006; Kissler et al., 2009; Frühholz et al., 2011; Rellecke et al., 2011). Although P1 component was not affected by emotional words, emotional stimuli did significantly modulate the subsequent ERP components. We observed that affectively negative words elicited a more negative N170 amplitude than positive and neutral words. The negativity bias (Carretié et al., 2001) which should appear in the earlier emotion processing was shifted toward the N170 component. Differences in the experimental tasks used in different studies may account for this discrepancy. Unlike emotion judgment tasks, the task employed in the present study facilitated only bottom-up influence on emotion processing. Thus, the time course of the negativity bias was shifted toward later ERP components. The orbitofrontal cortex could analyze coarse information fast which could modulate activity via the amygdala to alert potentially dangerous or disgust stimuli and generate quick responses. Thus, the negativity bias might be considered biologically adaptive.

An early logographic processing stage may be indicated by the N170 component for emotional words, and this stage could be affected by emotional value of words (Simon et al., 2007). However, a recent study reported by Scott et al. (2009) has shown that early modulatory effects only emerge when emotional words have high familiarity (Scott et al., 2009). In the current experiment, we employed unfamiliar words that appeared in a moderate level of frequency in common language. In addition, another recent study reported by Thomas et al. (2007) showed no emotion effects on the N170 component when using color naming and emotion judgment tasks (Thomas et al., 2007). Although this appears inconsistent with our findings, it may be that the availability of attentional resources strongly influences the N170 component, and the reported differences may thus be due to different experimental paradigms that provide correspondingly different attentional demands. In the present study, specific design aspects, such as limiting attentional resources, and the use of a moderate level of word appearance frequency, may have promoted our discovery of an emotionally sensitive early N170. The allocation of attention to negative words at this processing stage presumably occurred automatically due to the limits our task imposed on the participant’s ability to consciously redirect attention. However, although prior research has shown that the left hemisphere N170 component responds to emotional words, the present study did not demonstrate such a left hemisphere advantage. There may be two reasons for this. First, the task in the present study entailed participants detecting upright words within a stimulus train of inverted words, requiring only superficial grapheme analysis for success. In contrast, in an emotion judgment task, individuals must devote considerably more resources to successful emotion word processing in order to be successful. As such, the left hemisphere advantage was weakened in the present word detection study. Furthermore, our emotion-irrelevant task would involve more perceptual processing while emotion judgment tasks necessarily engage more semantic processing. Thomas et al. (2007) have demonstrated that it is easier to classify perceptual characteristics than semantic characteristics (Thomas et al., 2007). Another study reported by Hinojosa et al. (2010) also demonstrate that it is necessary to direct attention to the emotional content in some degree of linguistic processing during word processing (Hinojosa et al., 2010). Moreover, it is noteworthy that an atypical use of component terms emerged in the current experiment. The typical peak latency for N170 is 170 ms (Luck, 2014) while we used latencies in the 250–290 ms range (**Figure 2**). We defined components on the basis of scalp topography. The relatively late N170 component in the present experiment is probably attributable to the RSVP task using a relatively difficult task which requires subjects to discriminate whether a digit string is odd or even and then to detect the appearance of an upright word in a rapidly presented stimulus sequence (see also Kessler et al., 2005; Luo et al., 2013; Zhang et al., 2014 for a similar ERP latency delay).

### Late Effects of Word Emotion Type

In the processing of emotional pictures and words, a number of studies have shown that the LPP amplitudes elicited by stimuli with positive emotional valence on are likely to be similar to, or sometimes even larger than elicited by stimuli with negative valence (Frühholz et al., 2011; Zhang et al., 2014). With regard to emotional processing, the increased motivational significance of emotional stimuli is likely to modulate LPP effects (Kayser et al., 1997; Schupp et al., 2000; Eimer and Holmes, 2002). In contrast to previous results (Frühholz et al., 2011; Zhang et al., 2014), our current data suggest that the brain discriminates between negative and positive emotions via temporal dynamics. In a previous study (Luo et al., 2010), we found that the LPP component reflected the differentiation of three types of emotional words in a single time window. However, the present study showed that positive, neutral, and negative emotions were distinguished in two separate time windows. For instance, positive words evoked larger LPP amplitude than negative and neutral words within the 480–580 ms window, consistent with our previous study of emotional word processing, while negative words evoked larger LPP amplitudes than neutral words within the 580–680 ms window. The LPP amplitude evoked by positive words was thus larger than that for negative and neutral words, which is consistent with previous results. The reason why people transfer their focus from negative stimuli and positive stimuli is in later word processing stage positive words may promote the process of attentional capture, encoding and evaluation (Herbert et al., 2006). The latency delays observed for LPP are probably attributable to a relatively difficult task employed in this study, which does not provide any contextual information.

The processing advantage reflected in ERPs for affectively positive words found in the present study is consistent with previous literature (Schapkin et al., 2000; Herbert et al., 2006, 2008; Zhang et al., 2014). This advantage (the bias toward positive information resulting in deeper evaluation, encoding, and memory for positive relative to negative and neutral stimuli) for positive information has been termed the “positivity bias” (Cacioppo and Gardner, 1999). At this late temporal stage, the emotional value of words may also undergo further processing, even when it is not explicitly attended to (Kissler et al., 2009; Schacht and Sommer, 2009a,b).

Affectively positive and negative words evoked similar LPP amplitudes in the 580–680 ms time window, and these amplitudes were greater than those seen for affectively neutral words. This may demonstrate that an enhanced amplitude of the LPP for emotion relative to neutral words only appears when the words were deeply processed (Hinojosa et al., 2010). Consistent with previous research (Kissler et al., 2009), we found that the LPP was likely to be enhanced when processing emotionally positive and negative words compared to neutral words. When attentional resources are limited, the amplitude of the LPP decreases as task demands increase (Hagen et al., 2006). The present study found that more attentional resources were devoted to the emotional word processing, as demonstrated by relatively large LPP amplitudes for emotional versus neutral words. The LPP component might represent an underlying neural mechanism that distinguishes between emotional and neutral stimuli.

Prior evidence has suggested that the enhancement effect of the LPP in response to emotionally positive words is based in visual areas of the cortex. However, Herbert et al. (2006) found that electric potentials elicited by auditory positive words also showed a similar enhancement effect (Herbert et al., 2006). Furthermore, we suggest that individuals might not process the semantic information contained in words directly but might silently voice the word. Thus, semantic information was extracted more slowly in emotional-irrelevant than in emotion judgment tasks. On this basis, further research is needed to elucidate the role of phonetic processing in emotional word processing.

As shown above, the ERP dynamics observed in the present study, which used a word detection task, differed from those documented in a previous study that used an emotion judgment task. First, the P1 component, which reflected a negativity bias in our previous study, did not reflect such a bias in the present study. Compared to our current emotion-irrelevant task, the prior results may be due to top-down influences, which could provide a meaningful context for emotional processing. Prior studies promoted the automatic allocation of attention in the time period of P1. However, the current study does not necessarily indicate that the negativity bias was completely eliminated. We found that larger N170 amplitudes were elicited by negative words versus positive and neutral words, which demonstrates that the absence of a top-down influence on emotion processing led to a longer duration in processing emotion information. Second, we previously found that EPN components can be elicited by the visual presentation of words. Generally, the EPN was suggested to reflect semantic analysis in a task-independent fashion (Kissler et al., 2009; Schacht and Sommer, 2009b). The present study failed to show any difference between emotion extraction and semantic analysis, which is consistent with previous studies that used comparably superficial, perceptual, and emotion-unrelated tasks (Chwilla et al., 1995; West and Holcomb, 2002; Rellecke et al., 2011). Therefore, if the processing in word detection task was limited to detecting the changes in visual feature, an ERP effect of the adjectives emotional valence should not be detected, since it is supposed that attention to low-level features disrupts semantic activation in reading (Maxfield, 1997).

In summary, our current results showed that when the affective content of stimuli is task-irrelevant, the N170 component of the ERP is enhanced for affectively negative words, reflecting automatic processing of negative information. Moreover, the LPP was more sensitive to the emotional content with deeper processing. Specifically, the present study showed that positive, negative, and neutral emotional content were distinguishable in two different time windows. We suggest the brain shows a high degree of flexibility in processing emotional valence.

## Author Contributions

WZ, CZ, and WL conceived and designed the experimental paradigm, performed the experiments, and wrote the manuscript. WZ, CZ, LC, and WL reviewed and edited the manuscript. All the authors read and approved the manuscript.

## Conflict of Interest Statement

The authors declare that the research was conducted in the absence of any commercial or financial relationships that could be construed as a potential conflict of interest.
